# *Rhizobium sophorae* strain 33504-Borg2 as a biocontrol agent to mitigate the impacts of cucumber mosaic virus infection in faba bean

**DOI:** 10.3389/fpls.2025.1661085

**Published:** 2025-09-10

**Authors:** Ahmed Abdelkhalek, Karrar A. Hamzah, Toufic Elbeaino, Said I. Behiry, Hassan Moawad, Abdulaziz A. Al-Askar

**Affiliations:** ^1^ Plant Protection and Biomolecular Diagnosis Department, Arid Lands Cultivation Research Institute, City of Scientific Research and Technological Applications, Alexandria, Egypt; ^2^ Plant Protection Department, The National Institute of Horticultural Research, Skierniewice, Poland; ^3^ Department of Horticulture and Landscape Planning, College of Agriculture, Al-Qasim Green University, Babylon, Iraq; ^4^ Department of Integrated Pest Management of Fruit Trees and Vegetable Crops, Istituto Agronomico Mediterraneo di Bari, Bari, Italy; ^5^ Agricultural Botany Department, Faculty of Agriculture (Saba Basha), Alexandria University, Alexandria, Egypt; ^6^ Agriculture Microbiology Department, National Research Centre, Cairo, Egypt; ^7^ Department of Botany and Microbiology, College of Science, King Saud University, Riyadh, Saudi Arabia

**Keywords:** antioxidant, cucumber mosaic virus, faba bean, gene expression, polyphenolic compounds, *Rhizobium sophorae*

## Abstract

The application of *Rhizobium* spp., nitrogen-fixing bacteria, as a biocontrol agent to improve resistance to plant viral infections signifies a promising approach for sustainable and eco-friendly agricultural practices. The current study evaluated the effectiveness of strain 33504-Borg2 of *Rhizobium sophorae* in promoting faba bean growth and enhancing its systemic defense response to cucumber mosaic virus (CMV). Under greenhouse conditions, the pre-treatment of soil with 33504-Borg2 resulted in significant improvements in plant growth, an increase in chlorophyll content, as well as a reduction in the incidence and severity of disease and CMV accumulation by 44%, 72.5%, and 71%, respectively. The application of 33504-Borg2 inoculation also demonstrated a reduction in non-enzymatic markers of oxidative stress, such as hydrogen peroxide and malondialdehyde, alongside a notable increase in enzymes that mitigate reactive oxygen species, including polyphenol oxidase and peroxidase. Moreover, the expression levels of some genes associated with defense mechanisms, including pathogenesis-related proteins and those involved in the polyphenolic pathway, showed a notable increase. The HPLC analysis indicated that plants treated with 33504-Borg2 exhibited increased accumulation of numerous polyphenolic compounds, including gallic acid, ellagic acid, coumaric acid, pyrocatechol, and catechin. Hence, the capacity 33504-Borg2 to promote plant growth and enhance systemic resistance to CMV encourages its application as a biocontrol and biofertilizer agent. This application offers an innovative approach to protecting plants, promoting sustainability, and guaranteeing agricultural environmental security. To the best of our knowledge, this study represents the first investigation of *R. sophorae* as a biocontrol agent against CMV infestation.

## 1 Introduction

Faba bean, *Vicia faba* L., is a widely recognized grain legume valued for its high nutritional quality, serving both as a source of human food and animal feed (Bellof and Freitag, 2025). The primary agronomic advantage of the faba bean lies in its ability to enhance nitrogen levels in the system through biological nitrogen fixation (Barłóg et al., 2018). The area dedicated to the cultivation of this crop experienced a decline, shrinking to 2.6 million hectares in 2019 from 5.6 million hectares in 1961, despite its economic importance (Adhikari et al., 2021). This decline could be partly linked to crop losses resulting from the sensitivity of faba beans to both biotic and abiotic stresses. Plant viruses pose a considerable risk to food security, leading to outbreaks in all crops of economic significance (Khangarot et al., 2025). It is estimated that nearly 47% of new and re-emerging plant diseases worldwide are attributed to plant viruses (Manjunatha et al., 2022). Reports indicate that around 50 viruses considerably impact the productivity of faba beans (Yassin et al., 2024). The yield losses of faba bean attributed to these viruses vary between 5% and 20%, reaching as high as 90% in fields with severe infections (Li et al., 2024). Recent studies have documented the occurrence and spread of CMV across various legume crops (Ogunsola et al., 2025; Younis et al., 2025).

Cucumber mosaic virus (CMV, genus *Cucumovirus*, family *Bromoviridae*) is one of the most prevalent and economically important plant RNA viruses worldwide (Aseel et al., 2023). It exhibits a remarkably diverse host range, encompassing over 1,200 plant species across more than 100 families, including monocots and dicots (Jones and Congdon, 2024). CMV transmission occurs via seeds and is easily transmitted by sap; however, its rapid dissemination is frequently attributed to aphid vectors (Mueller et al., 2012). Although CMV ranks among the most prevalent viruses in cucurbits, increasing evidence suggests it also triggers epidemics in significant leguminous crops (Nyadzani Mushadu, 2023). Field investigations revealed that CMV reduced the shoot dry weight of lentils by 72 – 81% and seed production by 80 – 90% (Latham et al., 2004). Furthermore, it results in considerable yield losses in chickpea, estimated at 45%, when the incidence of CMV reaches 75% (Jones et al., 2008). Consequently, managing viral diseases is crucial for improving agricultural productivity and meeting the food demands of a growing population (Vishnoi and Goel, 2024). Several studies have demonstrated the significance of beneficial microbes, including plant growth-promoting rhizobacteria (PGPR), in protecting plants against viral infections (Abdelkhalek and Hafez, 2019; Khalid et al., 2025).

*Rhizobium* bacteria, a type of plant growth-promoting rhizobacteria, enhance soil fertility through symbiotic nitrogen fixation, thereby promoting legume plant growth and alleviating plant stress (Krishnan et al., 2025). Besides supplying symbiotic nitrogen, rhizobia exhibit beneficial physiological traits, including the production of plant growth-promoting phytohormones such as cytokinins, indole-3-acetic acid, gibberellins, and riboflavin (Mir et al., 2022). Such compounds play multiple roles in enhancing plant growth and boosting productivity (Saranraj et al., 2023; Singh et al., 2025). Induced Systemic Resistance (ISR) is a form of plant immunity that is activated by certain non-pathogenic microbes, including rhizobia, and is distinct from pathogen-triggered systemic acquired resistance (SAR). ISR is typically mediated through jasmonic acid (JA) and ethylene (ET) signaling pathways, rather than salicylic acid (SA), which is more associated with SAR. According to several reports, rhizobia can enhance the resistance of leguminous plants to pathogenic infections by triggering complex defense responses (Rabari et al., 2023). These responses include upregulation of defense-related genes, modulation of plant hormone signaling pathways (JA and ET), activation of physical barriers such as callose deposition and lignification, and even the induction of programmed cell death (PCD) at infection sites to limit pathogen spread (Rana et al., 2023). Additionally, rhizobia-induced ISR can prime the plant for faster and stronger activation of defense mechanisms upon subsequent pathogen attacks, a phenomenon known as “priming.” This multifaceted ISR response plays a crucial role in the overall health and productivity of legume crops under biotic stress (Basu et al., 2021; Yassin et al., 2024). Despite the limited focus on their potential as biocontrol agents, particularly in the realm of antiviral properties, certain strains of rhizobia may serve as effective biocontrol agents, potentially reducing the use of chemical pesticides in agricultural environments (Das et al., 2017; Kebede, 2021). Recent findings suggest that rhizobia exhibit antiviral properties by enhancing the plant’s defensive mechanisms (Abdelkhalek et al., 2023; Yassin et al., 2024). As a result, identifying new rhizobia biocontrol agents that are environmentally safe, capable of controlling plant viruses, and effective in nitrogen fixation is crucial for ensuring food security.

The current study aimed to isolate and evaluate the effectiveness of strain 33504-Borg2 of *R. sophorae* in enhancing the growth of faba beans and their capacity to resist CMV infection. We examined enzymes that eliminate reactive oxygen species (ROS), such as peroxidase (POX) and polyphenol oxidase (PPO), along with non-enzymatic indicators of oxidative stress, including hydrogen peroxide (H^-^O^-^) and malondialdehyde (MDA). Additionally, the expression levels of two pathogenesis-related protein genes {it}(PR - 1{/it} and *PR-2*) and four genes involved in the polyphenolic pathway, chalcone synthase (*CHS*), p-coumarate 3-hydroxylase (*C_3_H*), hydroxycinnamoyl Co-A shikimate hydroxycinnamoyl transferase (*HCT*), and cinnamate 4-hydroxylase (*C_4_H*), were assessed. The profile of polyphenolic compounds in the faba bean plant was effectively determined using high-performance liquid chromatography (HPLC) analysis.

## 2 Materials and methods

### 2.1 Source of virus inoculum and plant material

The virus-free seeds of the faba bean (*Vicia faba* L.) cultivar Saka, which is sensitive to CMV infection, were obtained from a certified supplier (Agriculture Research Center in Egypt) and were subjected to RT-PCR testing to confirm the absence of CMV and other viruses before planting. The purified CMV isolate Kh1, accession number OL348189, served as the viral source for inoculation (Abdelkhalek et al., 2022b).

### 2.2 Isolation and characterization of the most effective rhizobia isolate

The bacterial isolates were isolated from the root nodules of faba beans collected from local fields (30.828781, 29.618395) in Alexandria Governorate, Egypt. A 3% hydrogen peroxide solution combined with 70% ethanol was employed to disinfect the surface of the nodules. The yeast extract mannitol (YEM) agar medium was used to isolate rhizobial isolates on petri dish plates. A pure isolate was inoculated into a 50 mL sterile flask filled with YEM broth. The flask was subsequently positioned in an incubator and stirred for 48 hours at 150 rpm and 28°C. According to Somasegaran and Hoben (1994), faba bean plants were employed to evaluate the symbiotic effectiveness of the isolates. The rhizobia isolate demonstrating the highest symbiotic effectiveness with faba bean plants was chosen to evaluate its antiviral efficacy. The preliminary assessment of the antiviral efficacy of each isolate was performed on faba bean plants, which act as a systemic host for CMV (Abdelkhalek et al., 2023). The Wizard Genomic DNA Purification Kit was used to isolate the genomic DNA of the promising bacterial isolate (Promega Corporation, WI, USA). Primers (**Table 1**) targeting the *16S rRNA*, ATP synthase beta subunit (*atpD*), recombinase A (*recA*), N-acetylglucosaminyl transferase (*nodC*), and nitrogenase reductase Fe protein (*nifH*) genes were employed to identify the *Rhizobium* isolate at the molecular level. The PCR technique was performed as described previously (Gaunt et al., 2001; Laguerre et al., 2001; Yassin et al., 2024). The annotated sequences (1400 bp for *16S rRNA*, 480 bp for *atpD*, 495 bp for *recA*, 861 bp for *nodC*, and 720 bp for *nifH*) were deposited in GenBank and assigned the accession numbers PP917737, PX067936, PX067937, PX067938, and PX067939 for *16S rRNA*, *atpD*, *recA*, *nodC*, and *nifH*, respectively.

**Table 1:** Nucleotide sequences of primers used in this study.

Primer name	Abbreviation	Direction	Nucleotide Sequence (5′-….-3′)	References
16S ribosomal RNA	*16S rRNA*	Forward	AGAGTTTGATCCTGGCTCAG	(Heuer et al., 1997)
		Reverse	GGTTACCTTGTTACGACTT	
ATP synthase beta subunit	*atpD*	Forward	GCTSGGCCGCATCMTSAACGTC	(Gaunt et al., 2001)
		Reverse	GCCGACACTTCMGAACCNGCCTG	
Recombinase A	*recA*	Forward	TTCGGCAAGGGMTCGRTSATG	
		Reverse	ACATSACRCCGATCTTCATGC	
N-acetylglucosaminyl transferase	*nodC*	Forward	AYGTHGTYGAYGACGGATC	(Laguerre et al., 2001)
		Reverse	CGYGACAGCCANTCKCTATTG	
Nitrogenase reductase Fe protein	*nifH*	Forward	TACGGNAARGGSGGNATCGGCAA	
		Reverse	AGCATGTCYTCSAGYTCNTCCA	
Cucumber mosaic virus-coat protein	*CMV-CP*	Forward	GGATGCTTCTCCACGAG	Abdelkhalek et al. (2022a)
		Reverse	AGTGACTTCAGGCAGT	
Pathogenesis-related protein-1	*PR-1*	Forward	GTTCCTCCTTGCCACCTTC	(Kavroulakis et al., 2005)
		Reverse	TATGCACCCCCAGCATAGTT	
Endoglucanase	*PR-2*	Forward	TATAGCCGTTGGAAACGAAG	
		Reverse	CAACTTGCCATCACATTCTG	
Chalcone synthase	*CHS*	Forward	CACCGTGGAGGAGTATCGTAAGGC	(Rabie et al., 2024)
		Reverse	TGATCAACACAGTTGGAAGGCG	
*p*-coumarate 3-hydroxylase	*C3H*	Forward	TTGGTGGCTACGACATTCCTAAGG	(Abdelkhalek et al., 2018)
		Reverse	GGTCTGAACTCCAATGGGTTATTCC	
Hydroxycinnamoyl transferase	*HCT*	Forward	TCTCCAACCCCTTTTAACGAACC	
		Reverse	CAACTTGTCCTTCTACCACAGGGAA	
Cinnamate 4-hydroxylase	*C_4_H*	Forward	CCCAGTTTTTGAAATTGGCTTCA	
		Reverse	GCCCCATTCTAAGCAAGAGAACATC	
Elongation factor 1-alpha	*EF1-α*	Forward	GTGAAGCCCGGTATGCTTGT	(Mascia et al., 2010)
		Reverse	CTTGAGATCCTTGACTGCAACATT	
Beta-actin	*β-actin*	Forward	TGGCATACAAAGACAGGACAGCCT	
		Reverse	ACTCAATCCCAAGGCCAACAGAGA	

### 2.3 Multilocus sequence analysis and phylogenetic tree construction

The annotated *16S rRNA* gene sequence was analyzed to determine the percentage of similarity with closely related type strains identified in the latest version of the EzBioCloud *16S rRNA* database (https://www.ezbiocloud.net, accessed August 24, 2025) (Chalita et al., 2024). Since 16S rRNA gene sequence analysis does not provide sufficiently accurate results for determining species affiliation (Warabieda et al., 2023), multilocus sequence analysis (MLSA) was performed. The phylogenetic study employed the concatenated MLSA of two genes (*recA* and *atpD*), along with three additional genes (*16S rRNA*, *recA*, and *atpD*). Additionally, symbiotic genes, including the nodulation gene *nodC*, were analyzed independently. All phylogenetic trees were constructed using the MEGA12 software package employing the Maximum Likelihood technique, and bootstrap analysis with 500 replicate datasets was performed to assess the robustness of the clusters (Kumar et al., 2024). Evolutionary distances were computed utilizing the Tamura 3-parameter + G model for 16S rRNA phylogeny, the Tamura-Nei *+G +I* model for *atpD* + *recA* phylogeny, the Tamura-Nei *+G* model for *16S rRNA* + *atpD* + *recA* phylogeny, and the Tamura 3-parameter *+ I* model for *nodC* and *nifH* phylogenies, identified as the optimal models for nucleotide substitutions (Tamura, 1992).

### 2.4 Greenhouse experiment

A pot experiment was used to assess the effectiveness of the isolated *Rhizobium* for enhancing faba bean systemic resistance against CMV. The faba bean seeds were surface sterilized before being planted in pots measuring 40 cm in diameter with sand and clay sterilized in a comparable ratio (1:1). Following a 14-day growth period, similar-sized plants were chosen and then divided into four different treatment groups. Each group treatment had five biological replicates. There were five faba bean plants in each pot. The first treatment (control) involved faba bean plants that were mechanically inoculated with a viral inoculation buffer, while the soil was treated with sterile YEM broth. The second treatment (CMV) consisted of plants inoculated with CMV and soil treated with a sterile YEM broth. The third treatment (*Rhizobium*, 33504-Borg2) involved soil inoculated with a *Rhizobium* and plants treated with viral inoculation buffer. The fourth treatment involved soil inoculated with *Rhizobium* and plants inoculated with CMV (*Rhizobium* + virus, protective). For *Rhizobium* inoculation, the soil in each pot was inoculated with 2 mL of a 48 h-grown broth culture (1×10^9^ CFU/mL), while each non-Rhizobium pot received an equal volume of sterile YEM broth. For CMV treatments, the faba bean leaves were mechanically inoculated with 1 mL of a viral solution of 20 µg/mL of purified CMV inoculum (prepared in 10 mM phosphate buffer, pH 7.2, and 0.1% sodium sulfite) on the 20^th^ day of seed germination. Briefly, the two true upper leaves of each plant were treated with carborundum dust (600 mesh) and subsequently inoculated mechanically with the viral solution through gentle rubbing with a forefinger already soaked in the freshly prepared inoculum (Hafez et al., 2013). The pots were kept in an insect-proof, controlled greenhouse, with a consistent temperature of 28°C and 70% relative humidity. The plants were observed daily to assess the progression of symptoms. Samples were collected 23 days post-viral inoculation (dpi) for various analyses. Each pot was treated as an independent biological replicate, consisting of a total of fifteen leaves sourced from five distinct plants, three leaves from each plant. Each biological replicate underwent three technical replicates.

### 2.5 Assessment of chlorophyll, growth parameters, disease incidence & severity, and viral accumulation

AT 23 dpi, the length and weight of shoots and roots were determined, and leaf chlorophyll content was estimated by using the Chlorophyll Meter SPAD - 502 Plus (Konica Minolta, Inc., Tokyo). The disease incidence (DI) was assessed by the following calculation: DI (%) = the total number of infected plants divided by the total number of plants x 100. The disease severity (DS) of visual symptoms was assessed and scored according to Ogunsola et al (Ogunsola et al., 2021), with slight modifications. To monitor the DS, a rating system was implemented: 0 represents no symptoms, 1 represents a local lesion with chlorotic color and mild mosaic, 2 represents severe mosaic, and 3 represents deformations. The DS percentage is calculated by DS (%) = (disease scale × number of plants per scale)/(total number of plants × highest disease scale) × 100.

The *CMV-CP* accumulation levels were measured and evaluated using the real−time quantitative PCR (RT−qPCR) technique (**Table 1**).

### 2.6 Oxidative stress markers evaluation

Malondialdehyde (MDA), a marker of lipid peroxidation, and hydrogen peroxide (H_2_O_2_) were evaluated as markers of oxidative stress. For MDA, 10 mL of 5% trichloroacetic acid (TCA) was employed to grind 0.5 g of leaves, and the mixture was spun at 4000 rpm for 15 min. The supernatant suspension was mixed with an equal volume of 0.67 thiobarbituric acid solution (0.67%) and heated at 100°C for 15 min. After cooling, measurements of the absorbance were made at 600 and 532 nm. A measurement of MDA is mM/g FW (Heath and Packer, 2022). For H_2_O_2_, the modified potassium iodide method was used to assess its formation (Velikova et al., 2000). A 0.5 g sample of fresh leaves was ground in 5 mL of a 0.1% trichloroacetic acid (TCA) and centrifuged for 15 min at 12000 rpm. One mL of propidium iodide (100 mM) and 500 µL of the upper clear supernatant were mixed with 500 µL of phosphate buffer (10 mM). The amount of H_2_O_2_ was calculated as micromoles per gram of fresh weight (µmol/g FW). After five minutes, the absorbance of the reaction was measured at 390 nm at room temperature, using an H2O2 standard curve to calculate the results.

### 2.7 Antioxidant enzymes estimation

The plant extract was derived from dry, powdered plant material using four amounts of pH 7.0 phosphate buffer (100 mM), which also contained 1% w/v polyvinylpyrrolidone and 100 mM Na-EDTA. The clear supernatant was the source of peroxidase (POX) and polyphenol oxidase (PPO) activity. The activity of POX was assessed in a 1200 µL reaction volume by mixing guaiacol (5 mM, 500 µL) and H_2_O_2_ (1 mM, 120 µL) with 80 µL of plant extract. The absorbance was determined at 480 nm after a 10-minute incubation period at 30°C for the reaction. The activity of the POX enzyme was expressed as µM/g FW (Angelini et al., 1990). Regarding PPO, 1 mL of Quinone (50 mM in 100 mM Tris-HCl buffer, pH 6.0) was mixed with 500 mL of plant extract. After a 10-minute incubation at 25°C, the absorbance of the reaction mixture was determined at 420 nm. Under assay conditions equal to one unit of enzyme activity (mM/g fresh weight), the absorbance increased by 0.001 (Cho and Ahn, 1999).

### 2.8 Assessment of polyphenolic phytochemicals by HPLC analysis

Using an Agilent 1260 Infinity HPLC, variations in polyphenolic compounds, including flavonoids and phenolics, were examined across all groups (Behiry et al., 2023). Leaf samples from each group were collected, air-dried, and finely ground with an electric grinder. Two grams of grinding leaves were extracted in 20 mL of 98% ethanol by shaking in a water bath at 40°C for six hours. Following filtration with Whatman no.1 filter paper, the resultant solution was transferred to a new tube and concentrated using a rotary evaporator. A Zorbax Eclipse Plus C18 column (100mm x 4.6) was used. The mobile phase used in the HPLC system consisted of methanol, acetonitrile, and 0.2% phosphoric acid. A 20-mL injection volume was used for the sample. A VWD detector with a wavelength of 284 nm was used to quantify the levels of flavonoids and phenolic compounds. The HPLC system underwent calibration using standard compounds of known concentration and purity before analyzing the samples. A range of polyphenolic compounds was employed as standard compounds. These included vanillin, kaempferol, gallic acid, ferulic acid, hesperetin, catechin, pyrocatechol, ellagic acid, diazen, cinnamic acid, naringenin, apigenin, quercetin, syringic acid, rutin, and methyl gallate. The retention time of the identified compound was compared with that of the authentic standard compounds (Khamis et al., 2023). The chromatogram displayed the retention time and peak area of each compound, facilitating the identification and quantification of the components present in the sample.

### 2.9 Real-time quantitative PCR analysis

The transcriptional levels of four genes associated with the synthesis pathways of polyphenolic compounds (*CHS*, *C_3_H*, *C_4_H*, and *HCT*) and two pathogenesis-related genes (*PR-1* and *PR-2*) were evaluated through RT-qPCR analysis. Total RNA was extracted from fresh leaves using the guanidium isothiocyanate extraction method with some modifications (Abdelkhalek and Sanan-Mishra, 2019). Genomic DNA was eliminated by treating the extracted RNA with RNase-free DNase. The RNA concentration was measured using a nanodrop, and its integrity was assessed through agarose gel electrophoresis. Using 1 µg of RNA as a template, the RNA was transcribed into cDNA using random hexamer primers and the oligo (dT) reverse transcriptase enzyme Super-Script II (Invitrogen, Waltham, MA, USA), following the manufacturer’s instructions (Omar et al., 2022). The RT-qPCR was performed using a Qiagen device with a reaction volume of 25 µL. The reaction consisted of 12.5 µL of qPCR SYBR Green premix, 1 µL of each primer (**Table 1**), 9.5 µL of molecular-grade water, and 1 µL of cDNA. The reaction was cycled 40 times at 95°C for 15 seconds, 60°C for 30 seconds, and 72°C for 30 seconds, followed by a 3-minute linking step at 95°C (ElMorsi et al., 2015). The *EF1-α* and *β-actin* genes (**Table 1**) were employed as housekeeping reference genes to normalize gene expression levels. The accurate measurements and calculations of the relative transcription levels were performed using the 2^−ΔΔCt^ method (Livak and Schmittgen, 2001).

### 2.10 Statistical analysis

Statistical analyses were conducted utilizing CoStat software. An ANOVA was conducted to evaluate the overall effects of the treatments. A significance threshold of *p* ≤ 0.05 was established, and Tukey’s honest significant difference (HSD) test was used to identify significant differences among the treatment groups. The data are expressed as mean (M) with standard deviations (SD), represented as M ± SD. The significant difference among the groups was presented in alphabetical order, ranging from least important to most important (a > b > c), with identical letters indicating no significant difference. Values in the transcript that exceed 1 signify heightened levels of gene transcription, commonly referred to as upregulation. Values below 1 indicate downregulation, reflecting a decrease in transcriptional levels.

## 3 Results

### 3.1 Bacterial isolation and identification

**Figure 1:**
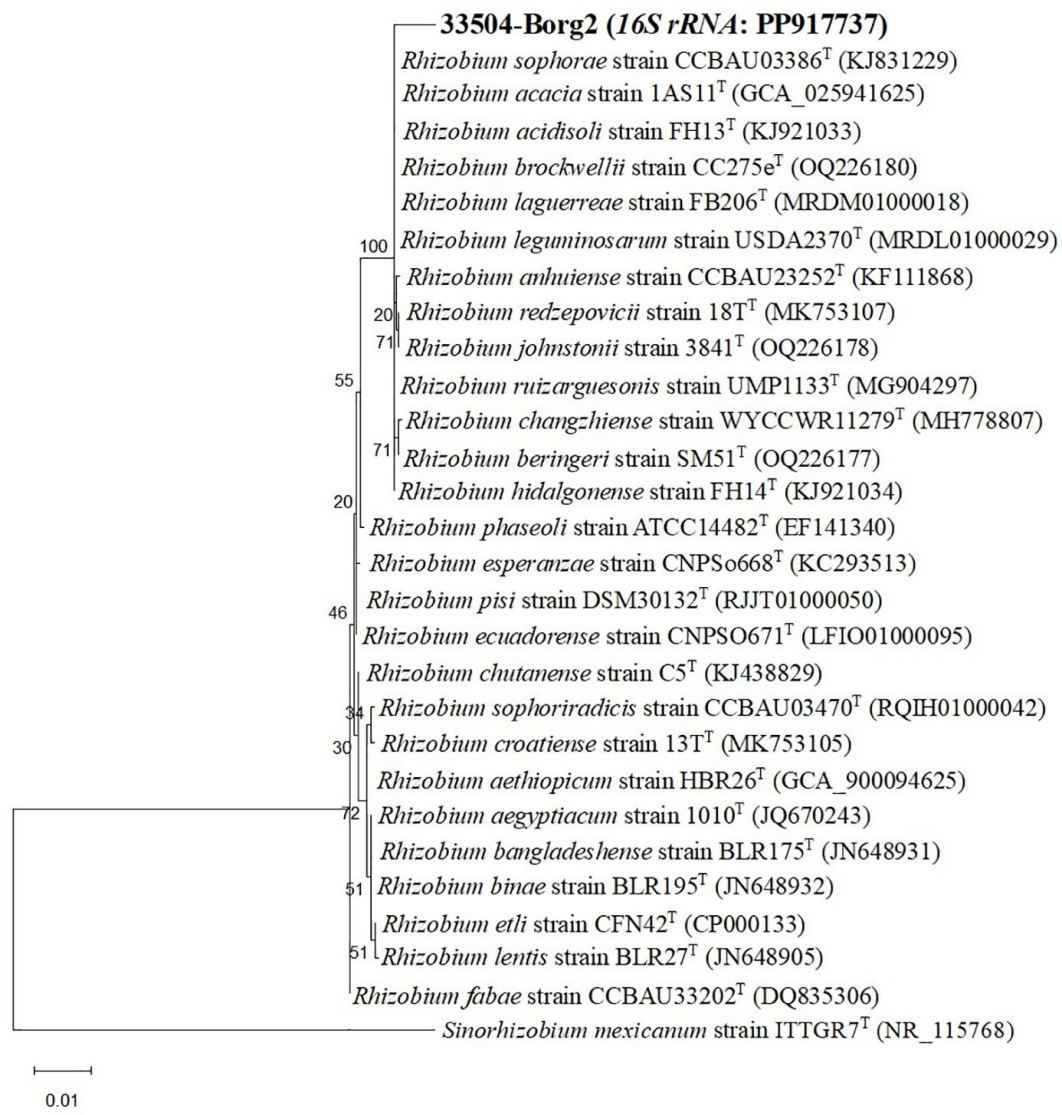
Maximum-likelihood phylogenetic tree based on *16S rRNA* gene sequences (1387 nt) showing the phylogenetic relationship of strain 33504-Borg2 (in bold) and related species of the genus *Rhizobium* under the best-fit model (Tamura 3-parameter *+G*). The branches display the percentage of replicate trees where the associated taxa clustered together (500 replicates). Evolutionary analyses were conducted in MEGA12 utilizing up to 4 parallel computing threads.

**Figure 2:**
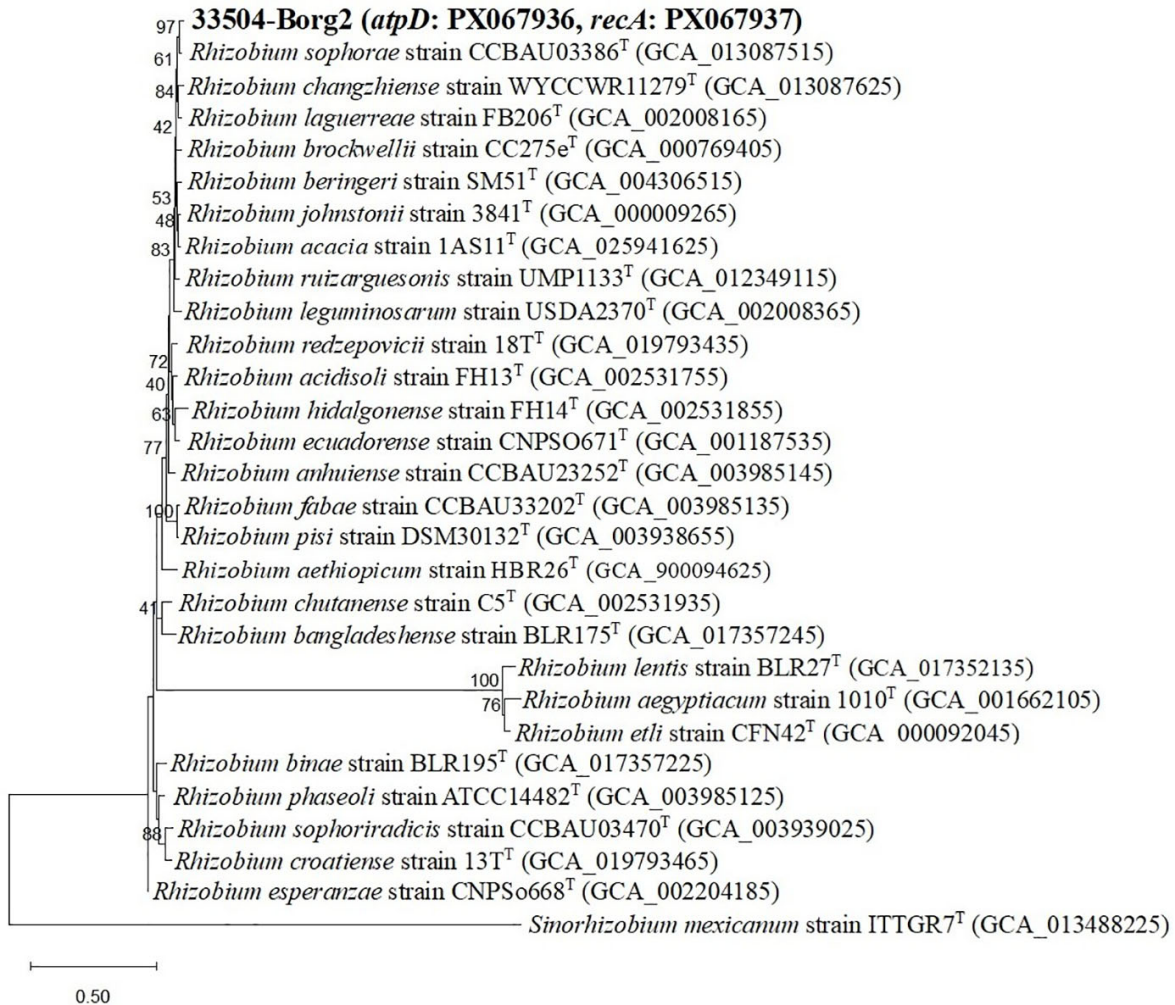
Maximum-likelihood phylogenetic tree derived from concatenated *recA* and *atpD* genes (961 nt) illustrating the relationship of the 33504-Borg2 strain (in bold) with other type strains within the genus *Rhizobium*. Bootstrap values derived from 500 replicates with the optimal model (Tamura-Nei + G + I) in the MEGA12 software.

**Figure 3:**
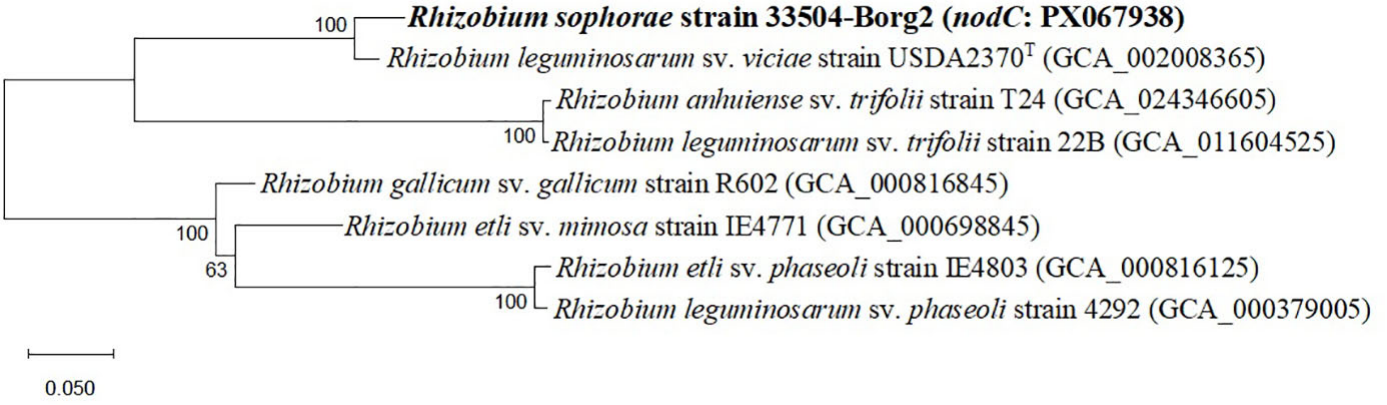
The maximum likelihood tree, derived from *nodC* gene sequence (861 nt), illustrates the phylogenetic relationships of *Rhizobium sophorae* strain 33504-Borg2 and related symbiovar species within the genus *Rhizobium*, utilizing the optimal model (Tamura 3-parameter + I). Bootstrap values, calculated from 500 replications, are displayed at the internodes.

Twenty-two rhizobial isolates were isolated and purified from the root nodules of the faba bean. The purified bacteria were Gram-negative, exhibited a rod-like cellular structure, and demonstrated a restricted ability to absorb Congo red. The isolate 33504-Borg2 exhibited the highest symbiotic effectiveness and antiviral activity; hence, it was chosen for identification and further use. The EzBioCloud dataset of bacterial type strains suggested that the rhizobial isolate be classified as *Rhizobium sophorae*. The analysis of the phylogenetic tree based on the nearly full-length *16S rRNA* gene region (1387 nt) indicated that the 33504-Borg2 strain shares a close relationship with *R. sophorae* strain CCBAU03386^T^, exhibiting approximately 98.5% similarity. This is followed by *R. acacia* strain 1AS11^T^ and *R. laguerreae* strain FB206^T^, both of which show around 98% similarity (**Figure 1**). Phylogenetic analyses utilizing sequences of housekeeping gene fragments *atpD* and *recA* (961 positions) indicated that strain 33504-Borg2 exhibited a high sequence similarity of approximately 98% to *R. sophorae* strain CCBAU03386^T^ (**Figure 2**). A similar topology was noted in the concatenated phylogenetic tree (2343 positions) derived from the three housekeeping genes (*16S rRNA*, *atpD*, and *recA*), where the 33504-Borg2 strain clustered with *R. sophorae* strain CCBAU03386^T^, demonstrating a sequence similarity of 98.5% (**Supplementary Figure S1**). The phylogenetic analysis of the nodulating gene, *nodC*, indicated that the 33504-Borg2 strain clustered with *R. leguminosarum* sv. *viciae* strain USDA2370^T^, exhibiting a similarity of 98% (**Figure 3**). A comparable topology was observed in the phylogenetic tree of the *nifH* gene, where the 33504-Borg2 strain clustered with *R. leguminosarum* sv. *viciae* strain USDA2370^T^, demonstrating a similarity of 100% (**Supplementary Figure S2**).

### 3.2 Faba bean growth parameters and the level of chlorophyll

The results of the present investigation (**Table 2**) indicated that CMV infection adversely affected both the dry and fresh weights, as well as the lengths of the shoots and roots, compared to the control plants. Conversely, applying *Rhizobium* 33504-Borg2 to the soil increased the weights and lengths of shoots and roots in comparison to the plants in the control (Mock). The protective treatment (33504-Borg2 + CMV) slightly mitigated the impact of viral infection on plant development, as evidenced by an increase in the dry and fresh weights of the shoots and roots compared to the plants treated with CMV only (**Table 2**). A similar effect was seen in the chlorophyll content of the plants. In the context of plant nodulation, it was observed that 33504-Borg2 treatment exhibited the highest number and size of root nodules. Although 33504-Borg2 induced nodulation under viral infection (protective treatment), a significant decrease in both the number and size of nodules was observed in infected faba bean plants compared to healthy ones (data not shown).

**Table 2:** Impact of *R. sophorae* strain 33504-Borg2 and CMV treatments on disease incidence, disease severity, virus accumulation level, growth parameters development, and antioxidant and oxidative stress markers of faba bean plants at 23 dpi.

Parameter		Treatments			
		Mock	CMV	33504-Borg2	Protective
Shoot	Length (cm)	36.3 ± 1.91 b	29.9 ± 1.54 d	41.3 ± 1.79 a	33.9 ± 1.39 c
	Fresh weight (g)	7.83 ± 0.98 b	6.61 ± 0.79 d	8.11 ± 0.89 a	7.28 ± 0.87 c
	Dry weight (g)	2.93 ± 0.75 b	2.07 ± 0.63 d	3.11 ± 0.73 a	2.68 ± 0.69 c
Root	Length (cm)	19.3 ± 1.45 c	14.8 ± 1.05 d	27.3 ± 1.53 a	20.1 ± 1.23 b
	Fresh weight (g)	6.17 ± 0.98 c	4.91 ± 0.84 d	7.21 ± 1.03 a	6.41 ± 0.97 b
	Dry weight (g)	2.16 ± 0.63 c	1.47 ± 0.54 c	2.87 ± 0.67 c	2.21 ± 0.49 c
Chlorophyll content (SPAD unit)		27.2 ± 1.39 b	19.7 ± 1.70 d	31.2 ± 1.99 a	26.3 ± 1.85 c
Disease incidence (%)		0 c	100 a	0 c	56 b
Disease severity (%)		00.0 ± 0.00 c	93.6 ± 3.68 a	00.0 ± 0.00 c	25.7 ± 2.03 b
Viral accumulation level		00.0 ± 0.00 c	25.8 ± 1.68 a	00.0 ± 0.00 c	7.32 ± 0.98 b
Hydrogen peroxide (H_2_O_2_)		7.23 ± 1.35 b	8.13 ± 1.30 a	7.34 ± 0.70 b	8.06 ± 0.89 a
Malondialdehyde (MDA)		146.7 ± 3.12 c	238.1 ± 1.24 a	92.2 ± 2.86 d	149.2 ± 5.01 b
Peroxidase		0.75 ± 0.01 b	0.57 ± 0.08 c	0.86 ± 0.03 a	0.73 ± 0.04 b
Polyphenol oxidase		0.53 ± 0.02 b	0.45 ± 0.01 d	0.57 ± 0.03 a	0.50 ± 0.01 c

Mock is a control (healthy plants); CMV is the viral treatment; 33504-Borg2 is the *Rhizobium* treatment; and protective is the *Rhizobium* + virus treatment. Statistical significance was indicated alphabetically in each column in ascending order, where a > b > c> d. Each column value represents the average of five biological replicates. The mean value of each column that shares the same letter is not significantly different as per Tukey’s HSD test (*p ≤* 0.05).

### 3.3 Impact of 33504-Borg2 on the disease incidence and severity, and viral accumulation level

**Figure 4:**
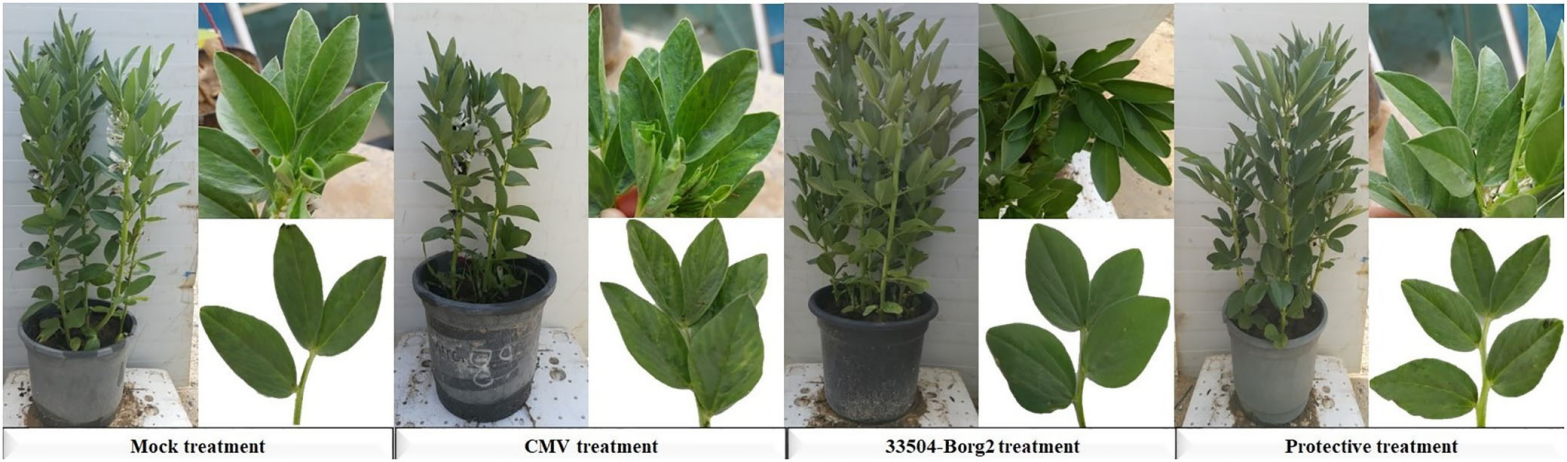
Impact of soil application of *R. sophorae* strain 33504-Borg2 on developing viral symptoms on faba bean leaves at 23 dpi. Mock: healthy control; CMV: faba bean inoculated with CMV only; 33504-Borg2: faba bean inoculated with 33504-Borg2 only; Protective: faba bean treated with 33504-Borg2 and then inoculated with CMV. The CMV treatment showed severe and apparent characteristic symptoms of CMV infection. The protective treatment showed mild viral symptoms, while the Mock and 33504-Borg2 treatments showed no symptoms.

The soil application of 33504-Borg2 resulted in a significant reduction in disease severity and a drop in CMV accumulation levels compared to plants infected only with CMV without treatment (**Figure 4**). The results indicated that the developed mosaic symptoms emerged following CMV infection at 16 dpi; however, the administration of 33504-Borg2 postponed symptom onset by five days, with mild symptoms manifesting at 23 dpi (**Figure 4**). No discernible symptoms were observed in either the Mock or 33504-Borg2 treatment groups. The CMV treatment demonstrated a 100% incidence of disease, with disease severity and *CMV-CP* accumulation levels at 93.6% and 25.8-fold, respectively (**Table 2**). The protective treatment reported a significant reduction in disease incidence and severity, achieving decreases of 56% and 25.7%, respectively. It also reduced the *CMV-CP* accumulation level by 7.32-fold. The noted reduction in viral accumulation is approximately 72%, indicating the ability of 33504-Borg2 to enhance faba bean plants’ suppression of viral replication and accumulation within plant tissues.

### 3.4 Impact of 33504-Borg2 on oxidative stress markers

The level of H_2_O_2_ was elevated in the CMV treatment compared to the Mock treatment, but was similar to the protective treatment with *Rhizobium*. Assessing MDA levels is a reliable method for detecting lipid peroxidation, a key biomarker of oxidative stress. The lipid peroxidation levels in the CMV group increased significantly to 238.1 mM/g f.wt., representing a 48% rise compared to the Mock treatment level of 146.7 mM/g f.wt. (**Table 2**). Treatment with only *Rhizobium* inoculation showed the lowest MDA values; however, the protective treatment demonstrated values similar to those with neither *Rhizobium* nor CMV inoculation.

### 3.5 Impact of 33504-Borg2 on the levels of antioxidant enzymes

The antioxidant activity of the two enzymes, peroxidase (POX) and polyphenol oxidase (PPO), was distinctly differentiated following CMV infection and 33504-Borg2 treatments (**Table 2**). The levels of peroxidase were slightly elevated in the plants treated with 33504-Borg2compared to the control plants. The CMV treatment exhibited the lowest value of peroxidase, whereas the protective treatment recorded a higher value, which was almost equal to that of the mock treatment but lower than *Rhizobium* inoculation alone. A similar trend in the values of polyphenol oxidase in the plants from different treatments was observed (**Table 2**).

### 3.6 Impact of 33504-Borg2 on the profile of polyphenolic compounds

**Figure 5:**
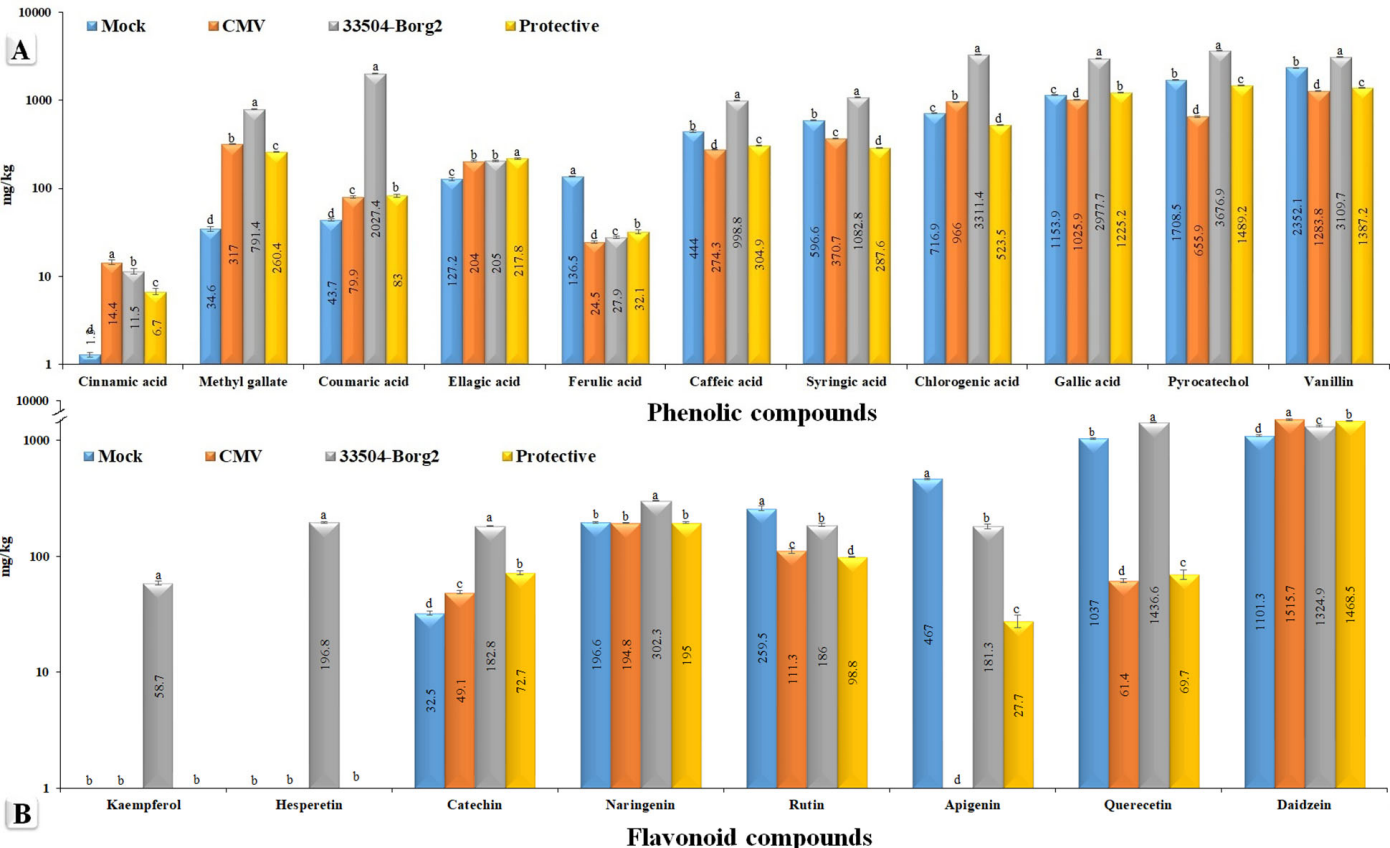
The variation of polyphenolic compound concentration (mg/kg) using high-performance liquid chromatography in the mock plants, CMV, 33504-Borg2, and protective treatments. Note: Mock is a control (healthy plants); CMV is the viral treatment; 33504-Borg2 is the *Rhizobium* treatment; and protective is the *Rhizobium* + virus treatment. Statistical significance was indicated alphabetically in each column in ascending order, where a > b > c > d. Each column value represents the average of five biological replicates. The mean value of each column that shares the same letter is not significantly different as per Tukey's H.S.D. test (*p ≤* 0.05).

HPLC analysis (**Supplementary Figure S3**) was conducted on the leaf samples collected at 23 dpi to evaluate the differences in phytochemical components across the various treatment groups of faba bean (**Figure 5**). The overall amounts of the 19 identified polyphenolic compounds were 10.409, 7.149, 21.885, and 7750 mg/kg in the Mock, CMV, 33504-Borg2, and protective treatments, respectively (**Figure 5**). The findings indicated a direct correlation between CMV infection and the 33504-Borg2 treatment, specifically in terms of the overall quantity of polyphenolic compounds, compared to control plants. The key compounds identified in the 33504-Borg2 treatment, listed in order of concentration, were 3676.9 mg/kg of coumaric acid, 3311.4 mg/kg of pyrocatechol, 3109.7 mg/kg of chlorogenic acid, 2977.7 mg/kg of vanillin, and 2027.4 mg/kg of gallic acid. Plants treated with 33504-Borg2 exclusively exhibited kaempferol and hesperetin, with concentrations of 58.7 and 196.8 mg/kg, respectively. In comparison to the mock treatment, the CMV treatment led to a notable reduction in the levels of gallic acid, caffeic acid, syringic acid, pyrocatechol, rutin, vanillin, ferulic acid, quercetin, and apigenin. Interestingly, the application of 33504-Borg2 as a protective treatment resulted in a significant enhancement in the synthesis of several compounds, including gallic acid, pyrocatechol, and vanillin, compared to the CMV treatment (**Figure 5**).

### 3.7 Impact of 33504-Borg2 on transcription levels of defense-related genes

**Figure 6:**
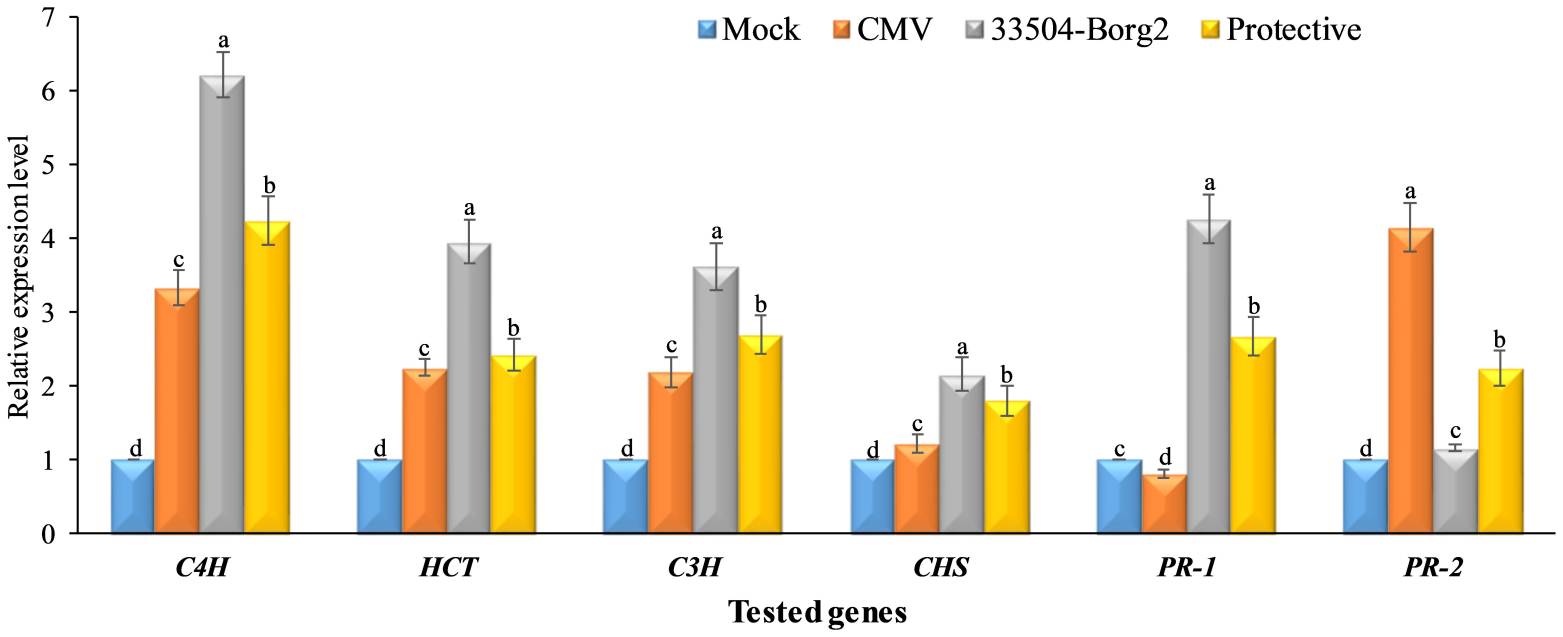
The expression levels of four polyphenolic genes (*CHS*, *C3H*, *C4H*, and *HCT*) and two pathogenesis-related genes (*PR-1* and *PR-2*). Differences between groups were determined using a one-way analysis of variance (ANOVA) and Tukey's HSD test at a significance level of *p* ≤ 0.05 in the CoStat statistical package. Statistical significance was indicated alphabetically above the histogram in ascending order, where a > b > c > d. The columns reflect the mean of five biological replicates. Columns with the same letter meaning do not differ significantly.

The results of the study regarding the expression levels of the four polyphenolic genes (*C4H*, *C3H*, *HCT*, and *CHS*) and two pathogenesis-related genes (*PR-1* and *PR-2*) (**Figure 6**). In the comparison of mock treatment, the CMV treatment showed a 3.33-fold increase in the transcriptional level of the *C4H* expression. Treatment with 33504-Borg2 showed the highest level (6.21 times) of *C4H* expression. The protective treatment showed a 4.24-fold increase, which was higher than in the mock and CMV treatments. The expression levels of the other three genes, *HCT, C3H*, and *CHS*, were higher in all three treatments than in the control. Nevertheless, the expression levels of *HCT* and *C3H* were lower than those of *C4H*, with *CHS* exhibiting the lowest levels. The 33504-Borg2 treatment resulted in a 4.26-fold increase in the expression level of the *PR-1* gene compared to the control plants (**Figure 6**). The CMV treatment showed the lowest level of *PR-1* expression. The protective treatment showed about 2.68-fold enhancement in the *PR-1* transcriptional level compared to the plants in the control and CMV treatments. The RT-qPCR results revealed low and nearly equal expression of the *PR-2* gene in plants from mock treatments and those inoculated with *Rhizobium* only. However, the expression of this gene in CMV-infected plants was significantly higher, approximately 4.14 times that of the plants in the mock treatment. This high value of expression was found to be reduced to almost half in plants from the treatment that received both CMV and *Rhizobium* inoculation.

## 4 Discussion

Plant diseases, particularly viral infections, cause significant agricultural losses worldwide, posing a threat to food security (Gai and Wang, 2024). There is an increasing emphasis on comprehending the role of rhizobia in mitigating both biotic and abiotic stressors. Nevertheless, reports on their effects on mitigating plant viruses are limited. Besides their ability to fix nitrogen in legumes, they contribute through the production of phytohormones, the reduction of ethylene levels in roots, and the secretion of specific compounds that enhance induced systemic resistance (Barquero et al., 2022; Fahde et al., 2023). Consequently, applying nitrogen-fixing *Rhizobium* for biocontrol is gaining recognition as an effective strategy for managing plant viral infections, presenting benefits compared to conventional chemical approaches in agriculture (Kebede, 2021; Abdelkhalek et al., 2023). In the current study, the antiviral properties of the *R. sophorae* strain 33504-Borg2 against CMV in faba bean plants were evaluated and reported for the first time. *R. sophorae* was first isolated from effective nodules of *Sophora flavescens* (Jiao et al., 2015) and has since been reported in nodules of other plants, including *Phaseolus vulgaris* and faba bean (El-Batanony et al., 2020; Zhang et al., 2022). The morphological characteristics of the 33504-Borg2 strain align with the descriptions provided for *Rhizobium*, as outlined in Bergey’s Manual of Systematic Bacteriology (Jordan, 1984). The analysis of the annotated *16S rRNA* sequence indicated that the 33504-Borg2 strain exhibited 98.5% similarity and clustered with *R. sophorae* CCBAU03386^T^, while also demonstrating sequence similarities of 97 - 98% with the type strains of other closely related *Rhizobium* species. The analysis of two housekeeping genes, *recA* and *atpD*, has been traditionally employed to distinguish closely related species within the genus *Rhizobium* (Gaunt et al., 2001). This is because 100% identity in *16S rRNA* gene sequences among rhizobial strains does not necessarily indicate that they are classified as the same species (Menéndez et al., 2024). Consequently, we conducted concatenated phylogenetic analyses utilizing the two genes from closely related *Rhizobium* type strains. The study of the concatenated genes revealed that 33504-Borg2 is phylogenetically closely related to the type strain of *R. sophorae* CCBAU03386^T^, indicating that it likely belongs to this species. In addition, *nodC* and *nifH* sequences exhibited high similarity to corresponding sequences of *R. leguminosarum* sv. *viciae* strain USDA2370T, suggesting that the 33504-Borg2 strain acquired its symbiotic genes through horizontal gene transfer. Recently, it was reported that several *R. sophorae* strains, isolated from faba bean nodules, acquired their *nodC* gene through lateral gene transfer from *R. leguminosarum* sv. *viciae* (Zhang et al., 2022).

Under greenhouse conditions, the soil application of *R. sophorae* strain 33504-Borg2 markedly improved the growth parameters of faba bean plants and increased the chlorophyll content compared to the CMV treatment. Moreover, it reduced the incidence of viral diseases by as much as 44% while decreasing severity by up to 72.5% and viral accumulation levels by up to 71%. These findings are consistent with previous studies that have reported direct effects of *Rhizobium* inoculation on the growth of various legume crops (Athul et al., 2022; Bahuguna et al., 2025; Jaiswal and Dakora, 2025). Furthermore, the diminished viral accumulation suggests that 33504-Borg2 may produce secondary metabolites that directly suppress CMV and/or significantly influence the activation of systemic acquired resistance (Boruah and Hage, 2024; Yassin et al., 2024). The collective action of antioxidant enzymes, including superoxide dismutase (SOD), peroxidase (POX), catalases (CAT), polyphenol oxidase (PPO), phenylalanine ammonia-lyase (PAL), and chitinase, is essential for effectively reducing oxidative stress and ensuring that free radical levels remain non-detrimental to plant health (Wu et al., 2017). Numerous investigations have shown that rhizobia stimulate the production of plant-related defense enzymes and antioxidants, potentially aiding the protection of plants against pathogens by triggering their defense mechanisms (Chen et al., 2015; Kong et al., 2015; Abdelkhalek et al., 2023). The findings indicate that CMV-infected faba bean treated with 33504-Borg2 (protective treatment) exhibited a notable enhancement in plant health, attributed to the substantial activation of POX and PPO. This evidence suggests that 33504-Borg2 plays a role in stimulating defense-related enzymes in the faba bean, reducing CMV infection. The findings align with earlier studies, which indicate a notable increase in antioxidant enzyme production in legume plants treated with *Rhizobium* sp (Hussain et al., 2024; Hernández et al., 2025). Several reports have indicated that virally infected plants generate increased levels of reactive oxygen species (ROS), resulting in oxidative stress (Mügge, 1998; Alazem and Burch-Smith, 2024). Our findings indicated a significant increase in oxidative stress markers MDA and H^-^O^-^ following CMV treatment. Notably, 33504-Borg2 assists plants infected with CMV by alleviating oxidative stress, evidenced by a substantial reduction in MDA and H^-^O^-^ levels. This improvement could be due to the interaction of 33504-Borg2 with infected plants, which induces defensive enzymes in the plant during viral stress, helping to maintain internal cell integrity by eliminating excessive ROS. The lowered H^-^O^-^ and MDA levels suggest that 33504-Borg2 efficiently mitigates membrane oxidative damage under viral stress.

Polyphenolic compounds are crucial secondary metabolites that enhance plant growth and protect against biotic and abiotic stresses (Aseel et al., 2025; Upadhyay et al., 2025). In the current study, CMV infection suppressed apigenin accumulation, likely weakening SA-mediated antiviral defense, as determined by HPLC analysis. This finding suggests that apigenin plays a significant role in the plant’s defense against viruses. Apigenin is a natural plant flavonoid that demonstrates significant antiviral efficacy against various viruses, including herpes simplex virus (Rittà et al., 2020), enterovirus 71 (Dai et al., 2019), and hepatitis C virus (Shibata et al., 2014). HPLC analysis indicated that CMV treatment significantly reduced the levels of vanillin, pyrocatechol, gallic acid, quercetin, syringic acid, caffeic acid, and rutin, which may be associated with the suppressive activity of CMV. Conversely, CMV treatment significantly increased the levels of daidzein, chlorogenic acid, methyl gallate, ellagic acid, catechin, and cinnamic acid, suggesting the potential antiviral efficacy of these compounds against CMV infection. It has been reported that methyl gallate exhibits various biological properties, including antioxidant and antimicrobial effects (Rahman et al., 2016). The application of 33504-Borg2 led to the induction and significant increase of various polyphenolic compounds, including vanillin, pyrocatechol, gallic acid, quercetin, apigenin, caffeic acid, ellagic acid, coumaric acid, and catechin, compared to CMV treatment. The findings suggest a hypothesis that these compounds may exhibit antiviral activity against CMV infection. Further investigation is required to validate this hypothesis. Coumaric acid exhibits multiple beneficial properties, including antioxidant (Pei et al., 2016), antimicrobial (Pragasam et al., 2013), and antiviral effects (Tanida et al., 2015). Gallic acid, a natural polyphenolic compound, exhibits antioxidant, antiviral, and antibacterial properties (Sethupathy et al., 2017; Bai et al., 2021). The HPLC analysis indicated that the 33504-Borg2 treatment resulted in the highest levels of vanillin, pyrocatechol, gallic acid, quercetin, chlorogenic acid, syringic acid, caffeic acid, naringenin, coumaric acid, methyl gallate, and catechin compared to other groups, suggesting that 33504-Borg2 functions as a PGPR. Notably, in contrast to the Mock, CMV, and protective treatments, the application of 33504-Borg2 resulted in the production of kaempferol and hesperetin. This observation indicates that 33504-Borg2 may enhance the production of novel compounds, acting as a supportive agent for growth promotion and stress tolerance. It was reported that the inoculation of rice plants with *R*. *leguminosarum* sv. *phaseoli* and *R. leguminosarum* sv. *trifolii* stimulated the synthesis of cinnamic acid, in contrast to uninoculated plants, which exhibited no presence of this compound (Mishra et al., 2006). Moreover, several volatile chemicals, including linalyl acetate, menthyl acetate, and α-farnesene, were exclusively identified in soybean plants infected with *Bradyrhizobium japonicum* sv *glycinearum*, in contrast to uninoculated plants (Couto et al., 2011).

The gene expression study revealed a significant increase in *C4H* expression following 33504-Borg2 treatment, with a relative expression level of 6.21-fold. This finding was followed by the protective and CMV treatments, which exhibited expression levels of 4.24-fold and 3.33-fold, respectively. *C4H* serves as an essential enzyme within the general phenylpropanoid pathway, catalyzing the conversion of cinnamic acid to *p*-coumaric acid, which is crucial for plant development, adaptation, and defense mechanisms against a range of biotic and abiotic stresses (Reinprecht et al., 2017). In line with RT-qPCR, HPLC analysis revealed that coumaric acid showed the highest accumulation in the 33504-Borg2 treatment, followed by protective and CMV treatments. As a result, the increased transcripts of *C4H* in 33504-Borg2 and/or CMV treatment indicate its role in defending against viral infections, suggesting that 33504-Borg2 could serve as a biocontrol agent to combat CMV infections by activating the biosynthesis of polyphenolic secondary metabolites in plant tissues (Yan et al., 2019). Similar to the *C4H* transcript profile, *HCT* and *C3H* genes exhibited the highest expression levels in the 33504-Borg2 treatment, followed by the protective and CMV treatments. *HCT* serves as the primary enzyme in the biosynthetic pathway of chlorogenic acid. It facilitates the conversion of *p*-coumaroyl CoA to shikimate (André et al., 2009). *C3H* subsequently converts shikimate into *p*-coumaroyl shikimate, forming chlorogenic acid (Akyol et al., 2016). The upregulation of transcriptional levels of these genes indicates their antiviral function, suggesting that intermediate compounds of the chlorogenic acid pathway may serve as a plant defense mechanism against viral infection, supporting systemic resistance (Kundu and Vadassery, 2019; Ramaroson et al., 2022). The HPLC analysis revealed an accumulation of chlorogenic acid pathway intermediate compounds in plant tissues, likely induced by SAR, which may enhance resistance to CMV infection (Bahar et al., 2021). *CHS* is a crucial enzyme that initiates the synthesis of *p*-coumaroyl-CoA in the flavonoid biosynthesis pathway of plants, leading to the formation of naringenin chalcone (Ferrer et al., 1999). This compound serves as a precursor for various flavonoid derivatives, including flavanols, flavones, glycosides, and anthocyanins (Zhang et al., 2017). The results indicated a significant increase in *CHS* expression in faba bean plant tissues due to CMV infection and/or *Rhizobium* treatments, with the 33504-Borg2 treatment demonstrating the highest level of expression. The findings align with Abdelkhalek et al. (2020), who noted that AMV infection triggers *CHS* in potato tissues. In contrast, the CMV-inoculated squash plants exhibited a reduced relative expression of *CHS* (Abdelkhalek et al., 2022b). Increasing *CHS* levels has been found to increase the amounts of flavonoid and isoflavonoid compounds, which possess properties that combat various plant diseases (Martínez et al., 2017).

Several studies indicate that pathogenesis-related (PR) proteins can trigger plant immune responses, contributing to SAR and inhibiting pathogen proliferation and/or dissemination (Simas et al., 2025). Our results indicated that CMV infection led to a significant decrease in *PR-1* expression, with a reduction of up to 20% relative to the mock treatment. Interestingly, the 33504-Borg2 and protective treatments significantly boosted *PR-1* levels, with the 33504-Borg2 group seeing an 80% increase compared to the CMV treatment. The findings align with the established function of the *PR-1* gene in reducing the impact of CMV and other mosaic virus infections (Abdelkhalek et al., 2022a, 2022b; Yassin et al., 2024; Salami et al., 2025). Salicylic acid (SA) is a recognized phytohormone molecule that serves as a plant signal, and its involvement in activating plant immunity has been documented for over twenty years. Moreover, numerous investigations have indicated that *PR-1* is a marker gene for salicylic acid, crucial in regulating systemic acquired resistance, and as a predictor for plant defensive responses (Javed et al., 2025). In the meantime, the induction of *PR-1* is often associated with the accumulation of SA, resulting in the activation of SAR (Tian et al., 2025). Consequently, we proposed that 33504-Borg2 could generate elicitor metabolite compounds that trigger systemic resistance, activating SAR and improving plant resilience against viral infections. Conversely, CMV infection significantly elevated *PR-2* in the CMV treatment, showing a relative transcriptional level 4.14-fold greater than that observed in the mock treatment. The results align with earlier studies that demonstrated notable activation of PR - 2 in response to viral infections across several plant species, including Arabidopsis, tobacco, potato, faba bean, tomato, and squash (Otulak-Kozieł et al., 2018; Abdelkhalek et al., 2022b, 2025). *PR-2* encodes a *β* - 1,3-glucanase protein, a crucial enzyme responsible for the degradation of the *β* - 1,3-glycosidic linkages in *β* - 1,3-glucans. This enzyme breaks down the callose deposits between plant cells, making its overexpression essential for most viruses to enable cell-to-cell viral movement and communication between cells (Kawakami et al., 2004; Oide et al., 2013; Otulak-Kozieł et al., 2018). Furthermore, diminished tobacco *PR-2* expression correlated with reduced susceptibility to viral infections, while its overexpression facilitated the rapid dissemination of viral infections among cells. Notably, the protective treatment applied to faba bean plants resulted in a substantial decrease in *PR-2* (2.25-fold) compared to the control plants. Consequently, applying 33504-Borg2 to the soil could potentially diminish CMV infection by decreasing *PR-2* expression and obstructing the long-distance movement of the virus between cells.

## 5 Conclusion

Under experimental greenhouse conditions, inoculating the soil with the *Rhizobium sophorae* strain 33504-Borg2 promoted faba bean growth and enhanced chlorophyll content following infection with CMV. Compared to CMV treatment, the protective treatment demonstrated a notable reduction in disease incidence, severity, and CMV accumulation by 44%, 72.5%, and 71%, respectively. The findings indicated a decrease in markers of non-enzymatic oxidative stress (H^-^O^-^ and MDA) while enhancing the activity of antioxidant enzymes (PPO and POX). Moreover, HPLC analysis also detected the accumulation of numerous polyphenolic compounds, including gallic acid, ellagic acid, coumaric acid, pyrocatechol, and catechin. In addition, it upregulated the expression of *PR-1* and polyphenolic pathway genes (*C4H*, *HCT*, *C3H*, and *CHS*). Overall, 33504-Borg2 promoted the growth of faba bean plants, enhanced systemic resistance, and exhibited significant antiviral properties against CMV. Consequently, 33504-Borg2 has the potential to serve effectively as a fertilizer and a preventive biocontrol agent, safeguarding plants against viral infections. However, further investigations are required to broaden the applicability of our results in field applications.

## Data Availability

The datasets presented in this study can be found in online repositories. The names of the repository/repositories and accession number(s) can be found in the article/**Supplementary Material**.
