# Supplementary material for: Rhizobium sophorae strain 33504-Borg2 as a biocontrol agent to mitigate the impacts of cucumber mosaic virus infection in faba bean
Source: Front Plant Sci. 2025 Sep 10;16:1661085. doi: 10.3389/fpls.2025.1661085 (PMC12457318; doi:10.3389/fpls.2025.1661085)
Supplement: Supplementary file 1 [file Supplementaryfile1.docx]

**
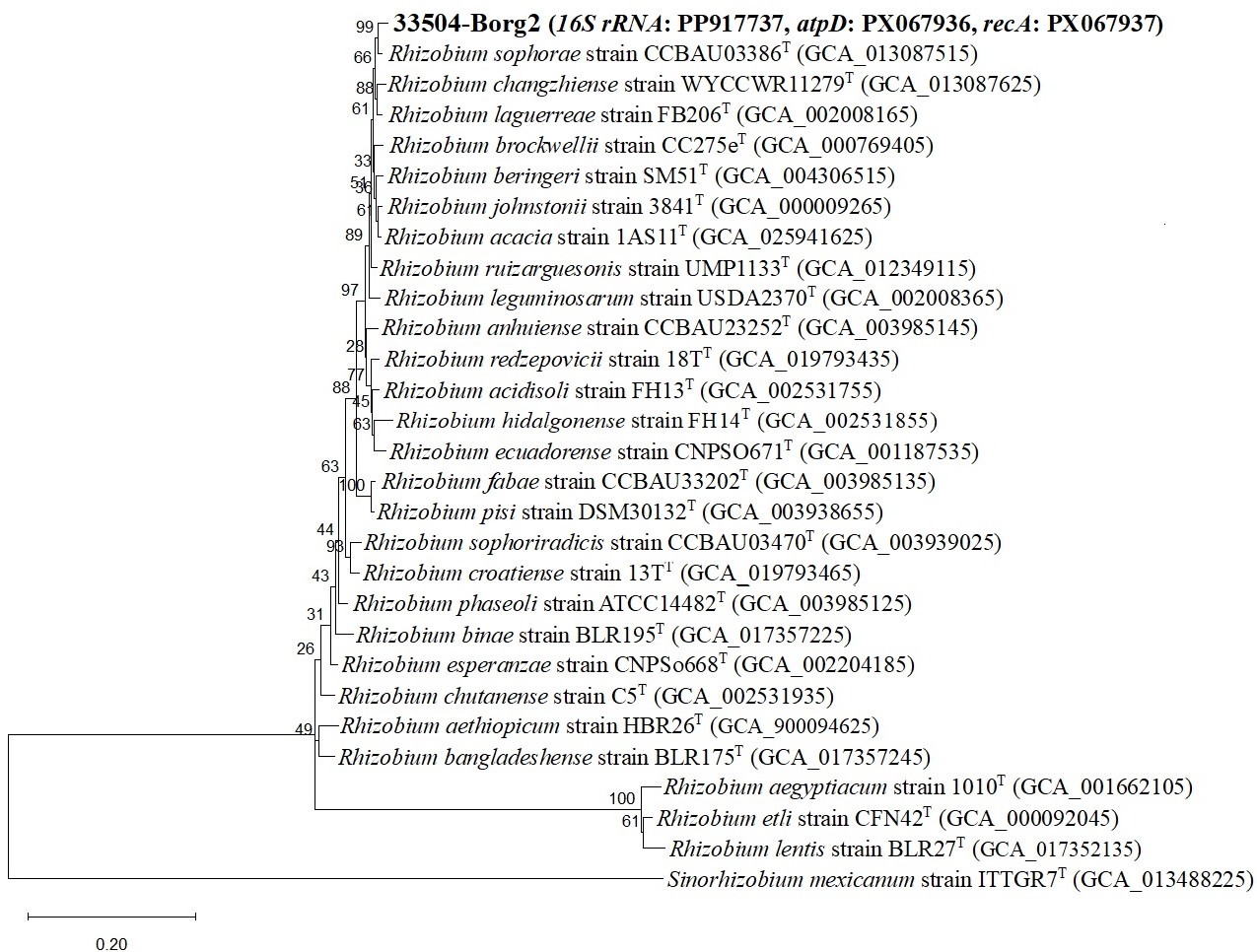
Figure S1.** A maximum likelihood phylogenetic tree was constructed using three housekeeping genes, which encompass a total of 2343 positions, to illustrate the relationships among *Rhizobium* *sophorae* strain 33504-Borg2 and closely related species within the genus *Rhizobium*. The genes were concatenated in the following order: *16S rRNA*, *atpD*, and *recA*. The percentage of replicate trees in which the associated taxa clustered together during the bootstrap test (500 replicates) is indicated next to the branches. Evolutionary distances were calculated using the Tamura-Nei model of nucleotide substitutions. The evolutionary rate differences among sites were modeled using a discrete Gamma distribution across 5 categories (*+G*, parameter = 0.3042), with 40.00% of sites deemed evolutionarily invariant (*+I*). Evolutionary analyses were conducted in MEGA12.

.

**Figure S2.** The maximum likelihood tree of *nifH* gene sequence (820 nt), illustrates the phylogenetic relationship of *Rhizobium* *sophorae* strain 33504-Borg2 and related symbiovar species within the genus *Rhizobium*, utilizing the optimal model (Tamura 3-parameter + I). Bootstrap values, calculated from 500 replications, are displayed at the internodes.


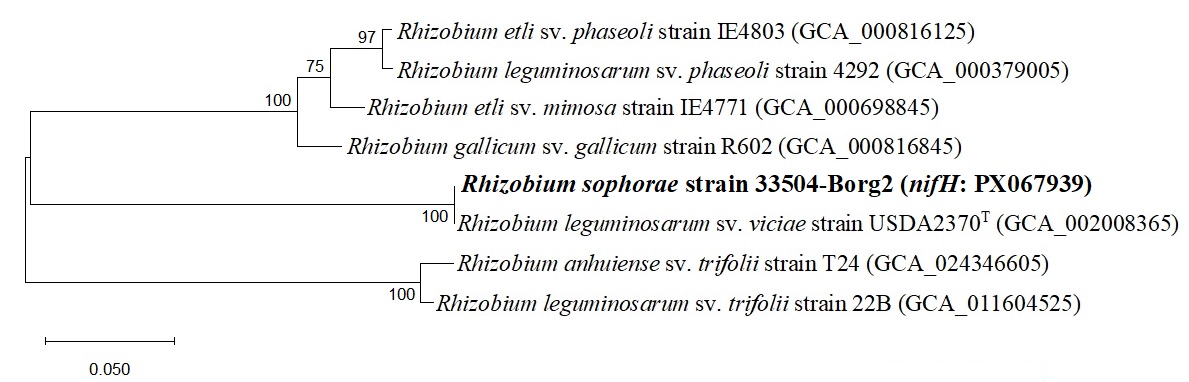


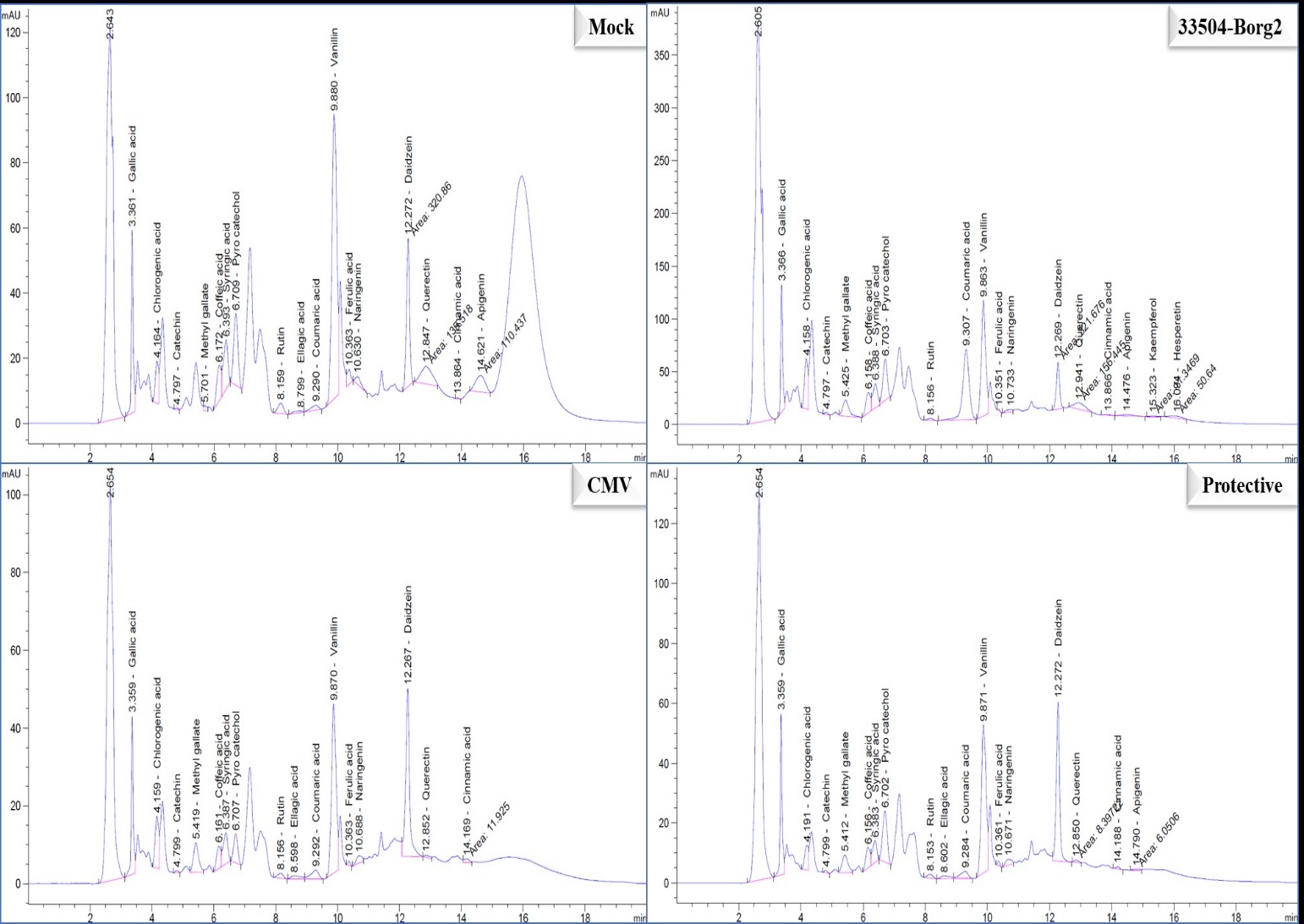


**Figure S3.** HPLC chromatograms of the polyphenolic compounds profile of the ethanolic extract of faba bean leaves at 23 dpi. Mock: healthy control; CMV: faba bean inoculated with CMV only; 33504-Borg2: faba bean inoculated with 33504-Borg2 only; Protective: faba bean treated with 33504-Borg2 and then inoculated with CMV.
